# Comparative Analysis of Growth and Feeding Behavior in Four Chinese Indigenous Pig Breeds

**DOI:** 10.3390/ani16152291

**Published:** 2026-07-23

**Authors:** Yu Chen, Xiaogang Zhao, Ji Zhu, Yingying Liu, Xionggui Hu, Huibo Ren, Huali Li, Lihua Cao, Qingming Cui, Yuan Deng, Weimin Jiang, Yinglin Peng, Chen Chen

**Affiliations:** 1 Hunan Institute of Animal and Veterinary Science, Changsha 410131, China; yuchen@hunaas.cn (Y.C.); zhaoxg@hunaas.cn (X.Z.); zhuji8717@hunaas.cn (J.Z.); hunaulyy_2025@hunaas.cn (Y.L.); huxionggui@hunaas.cn (X.H.); rhb526@hunaas.cn (H.R.); lihuali@hunaas.cn (H.L.); caolihua@hunaas.cn (L.C.); cuiqm5@hunaas.cn (Q.C.); dengyuan@hunaas.cn (Y.D.); jwm111@hunaas.cn (W.J.); 2 Yuelushan Laboratory, Changsha 410006, China

**Keywords:** indigenous pig breeds, growth performance, feed conversion ratio, feeding behavior, generalized additive mixed model

## Abstract

Indigenous pig breeds in China possess distinct biological characteristics, yet their growth dynamics and feeding behaviors remain inadequately characterized. This study compared growth performance, feed conversion ratio (FCR), and feeding behavior in four Hunan indigenous pig breeds, Dongshan (DS), Longtan (LT), Pushi (PS), and Taoyuan (TY), during the fattening stage (approximately 50–90 kg, over 60 days). Automated feeding stations were used to continuously monitor body weight, feed intake, and feeding behavior over 60 days in 48 pigs (12 per breed). Linear mixed model and generalized additive mixed model (GAMM) were employed to analyze growth trajectories, FCR dynamics, and temporal feeding patterns. The PS breed exhibited the highest daily gain (0.785 kg/day) and lowest FCR (3.98), whereas the DS breed showed the poorest feed efficiency (FCR 4.83). GAMM revealed significant non-linear changes in FCR and feeding rate across growth stages, with breed-specific critical windows of divergence. Notably, the TY breed achieved comparable growth to DS and LT with superior feed efficiency, suggesting adaptive metabolic characteristics. Diurnal feeding rhythms were conserved across breeds, despite marked differences in absolute feeding rates. These findings provide a fundamental database for precision feeding management and genetic improvement of indigenous pig breeds.

## Simple Summary

China is home to many traditional pig breeds that grow slowly but produce high-quality meat. Farmers and scientists need to understand how these pigs grow and eat to better protect and use them. This study compared four local pig breeds from Hunan Province over 60 days using automated feeding systems that tracked their weight, food intake, and eating behavior. The results showed that Pushi pig breed, grew the fastest and converted feed into body weight most efficiently. The Taoyuan pig breed exhibited the lowest average daily gain but achieved comparable final body weight to Dongshan (DS) and Longtan (LT) pigs with lower feed requirements per unit of weight gain, suggesting distinctive metabolic characteristics associated with efficient nutrient utilization. All breeds changed their feeding efficiency as they grew, with certain days being more critical than others. Interestingly, although some breeds ate faster than others, all pigs preferred to eat during the same daytime hours. These findings will help farmers develop better feeding strategies for each breed, reducing waste and improving production. They also provide valuable information for breeding programs aimed at preserving and improving China’s unique pig genetic resources.

## 1. Introduction

Indigenous pig breeds in China represent invaluable genetic resources shaped by millennia of natural selection and artificial domestication [1,2]. These breeds exhibit strong adaptability to local environments, superior meat quality traits, and remarkable genetic diversity, positioning them as strategic assets for ensuring the long-term sustainability of Chinese livestock production [3]. In contrast to commercial breeds selected for rapid growth and high lean meat percentage, Chinese indigenous breeds typically display distinct biological characteristics, including prolonged growth cycles, enhanced roughage tolerance, and elevated intramuscular fat deposition [4,5,6,7,8].

The Taoyuan (TY), Pushi (PS), Dongshan (DS), and Longtan (LT) pigs investigated in this study originate from different ecological regions of Hunan, representing important components of the indigenous pig genetic resources in southern China. TY and PS pigs are representative populations of the Xiangxi Black pig group, characterized by a uniform black coat, strong adaptability, and suitability for extensive production systems in western Hunan. TY pigs are characterized by large body size, high reproductive performance, and strong adaptability, whereas PS pigs exhibit compact body conformation and desirable meat quality characteristics [9]. DS and LT pigs belong to the Qianshao Spotted pig group originating from mountainous regions of southern Hunan. They display distinct spotted coat patterns, a more compact body structure and shorter tails, reflecting adaptation to mountainous production environments [10,11].

Recent large-scale genomic analyses have revealed pronounced population differentiation among Chinese indigenous pig populations, with genetic clusters largely corresponding to geographic distribution, local environmental adaptation and historical breeding [2,12,13]. Specifically, the 1000 Chinese Indigenous Pig Genomes Project, based on whole-genome sequencing of 1011 pigs from 50 indigenous populations, demonstrated clear genomic subdivision among Chinese local breeds, indicating that geographic isolation, historical demographic processes, limited gene flow, and long-term local selection have collectively shaped the genetic diversity of indigenous pigs [2].

Therefore, TY, PS, DS, and LT pigs provide a valuable model for investigating how genetic background influences growth performance and feeding efficiency under standardized environmental conditions.

To date, research on these indigenous breeds has largely concentrated on germplasm surveys, morphological characterization, meat quality evaluation, and reproductive performance assessment [7,8,14,15]. However, the systematic differences in growth patterns, feed efficiency, and feeding behaviors among these genetically distinct populations have yet to be thoroughly studied. The growth curve serves as a direct indicator of genotype-by-environment interactions, while feeding behaviors, including feeding rate and daily feeding patterns, represent key behavioral determinants of growth variation [16,17,18]. Traditional measurement approaches are constrained by their limited capacity for individual-level dynamic tracking. The development of automated electronic feeding stations has addressed this limitation by enabling precise, continuous monitoring of body weight gain, feed intake, and feeding behaviors throughout the fattening stage [19,20,21]. Despite these technological advances, studies on Chinese indigenous pig breeds mainly focus on static traits such as average daily gain and FCR calculated over the entire feeding period [22,23], rather than on dynamic growth trajectories or feeding behavior patterns [24,25,26]. The growth dynamics of these breeds, particularly how their performance varies across different developmental stages, remain poorly characterized. Addressing this knowledge gap is essential for conservation and sustainable utilization strategies.

Accordingly, this study employs an automated growth performance monitoring system to compare the growth patterns, FCR dynamics, and feeding behaviors of the four Hunan indigenous pig breeds, DS, TY, PS, and LT, during the fattening stage (approximately 50–90 kg, over 60 days). This study has three main objectives: to fit growth models for each breed and compare differences in average daily gain and feed conversion ratio; to characterize feeding behavior patterns, including feeding rate and diurnal rhythms; and to compare growth and feeding behavior data across breeds to identify stage-dependent patterns and explore potential associations between feeding behavior and growth performance. The findings aim to establish a baseline database for these indigenous breeds and provide a scientific foundation for breed-specific feeding management.

## 2. Materials and Methods

### 2.1. Experimental Animals and Feeding Management

Four indigenous pig breeds (DS, TY, PS, LT) were selected for this experiment. Each breed comprised 12 pigs (6 barrows and 6 sprayed gilts), resulting in a total of 48 experimental animals. All pigs were approximately the same age at the beginning of the experiment. Pigs were housed in a temperature- and humidity-controlled facility throughout the experimental period. For each breed, pigs of the same sex were housed together in a separate pen (6 pigs per pen), resulting in two pens per breed (one pen for barrows and one pen for spayed gilts). All pens were identical in size (2.8 m × 6.6 m) and equipped with fully slatted floors, automatic drinkers, and electronic feeding stations (Runnong Technology Co., Ltd., Shenzhen, China). The ambient temperature was maintained at 20–24 °C under a 12 h light/12 h dark cycle. All pigs had *ad libitum* access to feed and water throughout the experiment.

All pigs were fed a basal diet (Table 1) formulated to meet nutritional requirements for their growth stage. Feeding was conducted using automated feeding stations, which recorded daily body weight, total feed intake, feeding rate, and feeding time for each individual pig. Throughout the experimental period, all pigs were housed under standardized environmental conditions with appropriate temperature and humidity and *ad libitum* access to feed.

This study employed a balanced experimental design with approximately 60 days of continuous body weight monitoring (Table 2). A total of 2870 valid observations were obtained, with good observation integrity across breeds (DS: 60.9 days, LT: 59.0 days, PS: 60.8 days, TY: 58.5 days).

### 2.2. FCR Calculation and Time Window Optimization

Raw data were exported from the automated feeding system and subsequently organized. The first recorded date for each pig served as the reference for calculating relative days. Abnormal records and missing values were then screened, and valid observational data were retained for subsequent analyses.

To evaluate the influence of varying time window lengths on the stability of FCR estimation, six calculation schemes (7, 10, 14, 17, 21, and 30 days) were compared. FCR was defined as the ratio of total feed intake to total weight gain within each time window. For each window, linear models (LM) and linear mixed model (LMM) were fitted separately, and the optimal time window was determined by comparing model explanatory power (R^2^ and adjusted R^2^). Based on model performance, subsequent analyses employed the 21-day window for FCR calculation, yielding 129 valid FCR observations derived from repeated measurements of 48 pigs across three consecutive growth intervals: days 0–20 (Interval 0), days 21–41 (Interval 1), and days 42–60 (Interval 2).

### 2.3. Statistical Models

To comprehensively dissect the effects of breed, growth stage, and individual variability on various indicators, a progressive statistical modeling approach was adopted.

To analyze the fixed effects of breed on each indicator while accounting for repeated measurements within individuals, a LMM was established:

$$Y_{ijkl}=\mu +{\mathrm{Breed}}_{i}+{\mathrm{Sex}}_{j}+{\mathrm{Day}}_{k}+u_{l}+\varepsilon_{ijkl}$$

where $Y_{ijkl}$ is the observation for the i-th breed, j-th sex, k-th day, and l-th pig; μ is the overall mean; $breed_{i}$ is the fixed effect of the i-th breed; $Sex_{j}$ is the fixed effect of j-th sex; $Day_{k}$ is the fixed effect of experimental day k; $u_{l}∼N\left(0,\sigma_{u}^{2}\right)$ is the random effect of individual; and $\epsilon_{ijkl}∼N\left(0,\sigma^{2}\right)$ is the residual error.

Model fitting was performed using the lmer function in R, and the significance of breed effects (*p*-values) was extracted via Type II χ^2^ tests (car::Anova). For significant breed effects, Tukey’s post hoc multiple comparisons were conducted to identify pairwise differences with *p* < 0.05.

To capture non-linear temporal trends in the indicators, a generalized additive mixed model (GAMM) was employed:

$$Y_{ijkl}=\mu +breed_{i}+Sex_{j}+s\left(Day_{k},by=breed_{i}\right)+u_{l}+\varepsilon_{ijkl}$$

where $s\left(Day_{k},by=breed_{i}\right)$ is a breed-specific smooth function used to fit non-linear daily trends. Sex status was included as an additional fixed effect to account for potential differences between barrows and spayed gilts. The random effect $u_{l}$ accounts for correlation due to repeated measurements within individuals, and residuals $\varepsilon_{ijkl}$ are assumed to be independently and identically distributed.

To account for temporal autocorrelation arising from repeated measurements within individual pigs, several correlation structures were evaluated within the GAMM framework, including first-order autoregressive (AR1), compound symmetry (CS), continuous-time autoregressive (CAR1), and autoregressive moving average [ARMA(1,1)] structures. The optimal correlation structure was selected based on Akaike Information Criterion (AIC) and likelihood ratio tests. The ARMA(1,1) structure provided the best model fit and was applied in the final GAMM.

GAMMs were fitted using the gamm4 package, and the selection of smooth functions controlled for overfitting by limiting the degrees of freedom. Significant differences in smooth terms were determined by confidence intervals that did not cross zero, with the criterion for significant difference defined as: CI lower > 0 or CI upper < 0.

Sex (barrows and spayed gilts) was initially included as a fixed effect but was subsequently removed from the final models when it was found to be consistently non-significant (*p* > 0.05 in all analyses) and did not improve model fit (ΔAIC < 2). This decision was made to maintain model parsimony and interpretability, following established model selection principles [28].

### 2.4. Software and Statistical Tools

Data processing, statistical analysis, and visualization were all performed in the R environment (version 4.5.1) [29]. A significance level of α = 0.05 was used throughout, and all reported intervals are 95% confidence intervals. Data cleaning utilized the dplyr (version 1.8.9) [30], lubridate (version 1.9.5) [31], and purr (version 1.2.2) [32] packages, while visualization employed ggplot2 (version 4.0.3) [33], gratia (version 0.11.1) [34], and cowplot (version 1.2.0) [35]. Linear mixed models were fitted with lme4 (lmer function version 1.1.37) [36], with fixed effects tested via type II Wald χ^2^ tests (car::Anova) and post hoc comparisons using Tukey’s test (emmeans, version 2.0.0) [37]. Generalized additive mixed models were fitted using the gamm4 package (version 0.2.7) [38]. Model comparisons were based on AIC and likelihood ratio tests.

## 3. Results

### 3.1. Comparison of Growth Performance and FCR Variation Patterns Among DS, LT, PS, and TY Pig Breeds

**Figure 1:**
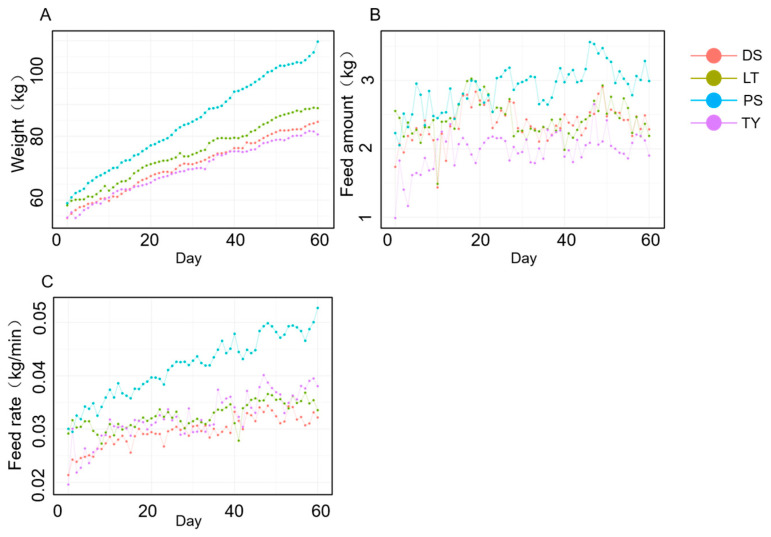
Daily records of body weight, daily feed intake, and feeding rate in DS, LT, PS, and TY pigs over the 60-day experimental period. (**A**) Body weight showed a general increasing trend across all four indigenous breeds throughout the 60 days. (**B**) Daily feed intake exhibited fluctuations over the experimental period. (**C**) Feeding rate progressively increased with time in all four pig breeds.

At the start of the experiment, the initial body weights (kg) of DS, LT, PS, and TY pigs were 54.36 ± 3.91, 58.33 ± 6.28, 59.01 ± 6.61, and 54.50 ± 7.68, respectively. No statistically significant differences in initial body weight were observed among the four indigenous breeds (*p* = 0.151). Over the experimental period, distinct growth trajectories were evident across breeds (Figure 1). Based on 60-day longitudinal body weight monitoring data, linear growth models were constructed for each breed (Table 3). The results indicated significant differences in growth rates among breeds (*p* < 0.001). Estimated daily gains are presented in Table 3. The PS breed exhibited the highest daily gain at 0.785 kg/day (95% CI: 0.709–0.861 kg/day), significantly surpassing the other three breeds (*p* < 0.001). Daily gains for LT, DS, and TY pigs were 0.534 kg/day (95% CI: 0.458–0.610 kg/day), 0.485 kg/day (95% CI: 0.409–0.561 kg/day), and 0.450 kg/day (95% CI: 0.374–0.526 kg/day), respectively. Following Tukey’s correction, differences among LT, DS, and TY breeds were not statistically significant (*p* > 0.05). The root mean square error (RMSE) of the model predictions was 2.011 kg, with a relative error rate of 2.68%, indicating a small prediction error. The standardized error metric (RMSE/SD) was 0.145, suggesting that the prediction error was considerably lower than the overall level of variation. The marginal R^2^ was 0.697, indicating that fixed effects accounted for 69.7% of the variation in body weight, while the conditional R^2^ was 0.979, demonstrating that the inclusion of random effects raised the total explanatory power for body weight variation to 97.9%.

To evaluate the influence of varying time window lengths on the stability of FCR estimation, six calculation schemes (7, 10, 14, 17, 21, and 30 days) were compared (Table 4). LM and LMMs with identical structures were fitted across different observation windows to evaluate the optimal period for FCR estimation. Results showed that as the time window extended from 7 to 21 days, the explanatory power (R^2^) of both LM and LMM generally increased, suggesting that longer observation periods enhance the stability of FCR estimates and model explanatory capacity. However, when the window extended to 30 days, model R^2^ declined. This phenomenon may be attributed to the reduced number of observations and available time points (87 observations and 2 time points), which may have limited model estimation, rather than reflecting changes in the biological properties of FCR.

Among all tested windows, the 21-day period showed the best balance between model performance and data availability, achieving the highest explanatory power (adjusted R^2^ = 0.457 for LM and conditional R^2^ = 0.506 for LMM). Thus, subsequent analyses on FCR calculation were based on the 21-day time window.

**Figure 2:**
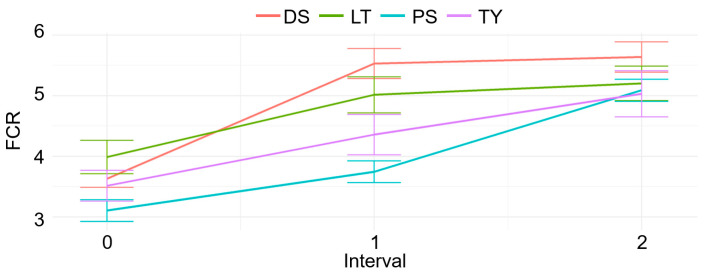
Distribution of FCR values calculated using the 21-day time window across three consecutive growth intervals for DS, LT, PS, and TY pig breeds. Interval 0: days 0–20. Interval 1: days 21–41. Interval 2: days 42–60.

Based on the 21-day window, a total of 129 valid FCR observations were obtained from repeated measurements of 48 pigs across three consecutive growth intervals: days 0–20 (Interval 0), days 21–41 (Interval 1), and days 42–60 (Interval 2) (Figure 2). Descriptive FCR statistics for each breed indicated that the DS breed had the highest mean FCR (4.83 ± 1.17), followed by LT (4.65 ± 1.04), while TY (4.27 ± 1.22) and PS (3.98 ± 1.04) breeds exhibited relatively lower values.

To comprehensively dissect the effects of breed, sex, growth stage, and individual variability on FCR, three progressive statistical models were employed. First, a LM served as the baseline to evaluate the fixed effects of breed, sex and stage. Second, a LMM incorporated individual random intercepts to account for the non-independence of repeated measurements within individuals. Third, a GAMM analysis further relaxed the linearity assumption to explore potential non-linear trajectories of FCR over time (Table 5). Results from all three models demonstrated that breed exerts a stable and significant influence on FCR. Compared to the DS breed, the PS breed showed significantly reduced FCR across all models (*p* < 0.001), and the TY breed also exhibited significantly reduced FCR (*p* < 0.05). Differences between the LT and DS breeds were not significant in any model (*p* > 0.05). Time effects were highly significant in both LM and LMMs (*p* < 0.001) (Table 5). Without accounting for individual random effects, FCR showed a more pronounced increase over time; after controlling for individual differences, growth stage remained a key determinant of FCR, with part of the temporal trend attributable to inter-individual variability. Sex effect on FCR was not significant, indicating that the breed-associated differences in feed efficiency were not explained by sex composition.

**Figure 3:**
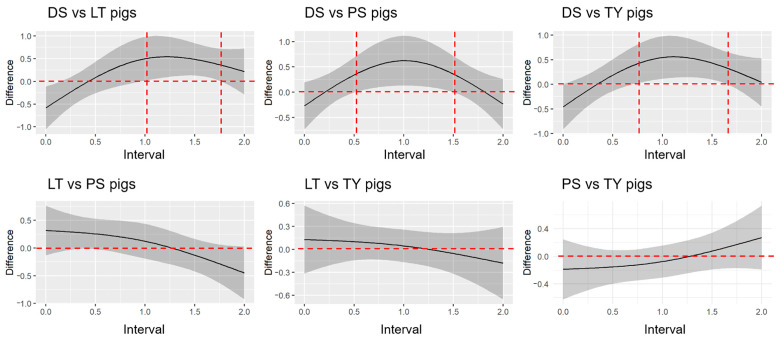
Temporal dynamics of FCR across growth stages for DS, LT, PS, and TY pig breeds as revealed by GAMM analysis. During the mid-growth phase, the DS breed consistently exhibited higher FCR compared to the other three breeds, although the specific time intervals of significant divergence varied by comparator. Interval 0: days 0–20. Interval 1: days 21–41. Interval 2: days 42–60. Shaded areas indicate 95% confidence intervals.

The GAMM analysis revealed that FCR changed significantly across growth stages for all breeds (*p* < 0.001), with notable differences among breeds. Sex was initially included as a fixed effect in the GAMM but was not significant (*p* = 0.195) and did not improve model fit; therefore, it was excluded from the final model to maintain parsimony and interpretability. Overall, during the mid-growth phase, the DS breed consistently exhibited higher FCR compared to the other breeds; however, the specific time intervals during which significant differences occurred varied slightly depending on the comparator breed (Figure 3). Specifically, the FCR of the DS breed was significantly higher than that of the LT breed between day 21 and day 36, higher than that of the PS breed between day 10 and day 31, and higher than that of the TY breed between day 15 and day 33 (Figure 3).

### 3.2. Comparison of Feeding Behavior Differences Among DS, LT, PS, and TY Pig Breeds

To evaluate the feeding behavior characteristics of different indigenous pig breeds, GAMM was employed to analyze the temporal dynamics of feeding rate (kg/min), with sex as a fixed effect and individual included as a random effect. Model comparison (Table 6) indicated that the full model (AIC = −22628), which incorporated breed effects and breed-specific smooth functions of time, provided a significantly better fit than the reduced model containing sex and individual effects only (AIC = −22557). The likelihood ratio test further confirmed the superiority of the full model (χ^2^ = 93.7, df = 11, *p* < 0.001). This suggests that breed differences and temporal trajectories contributed significantly to variation in feeding rate beyond sex and individual heterogeneity. The adjusted coefficient of determination for the full model was 0.354, explaining 35.4% of the variation in feeding rate.

Further parameter analysis (Table 7) revealed that, with DS breed as the reference, the baseline feeding rate of the PS breed was significantly higher (*p* < 0.001), representing a 40.5% increase compared to DS. In contrast, no significant differences in baseline feeding rate were observed for the LT breed (*p* = 0.314) or the TY breed (*p* = 0.372) relative to DS. The effect of sex on feeding rate was also not significant (*p* = 0.325).

**Figure 4:**
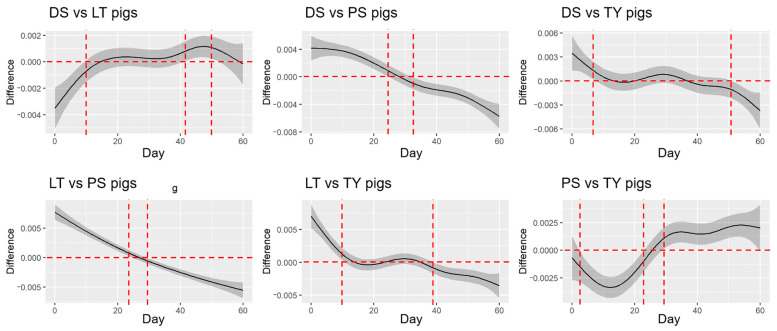
Stage-dependent changes in feeding rate among DS, LT, PS, and TY pig breeds over the 60-day experimental period, as estimated by GAMM analysis. Relative to the PS breed, DS, LT, and TY breeds exhibited higher feeding rates during the first 20 days but lower rates during days 30–60. Compared to LT breed, DS breed showed a lower feeding rate during the first 10 days but a higher rate during days 40–50. Relative to TY breed, DS breed had a higher feeding rate during the first 10 days but a lower rate during days 50–60. Similar stage-dependent reversals were observed between LT and TY: LT breed showed a higher feeding rate during days 0–10 but a lower rate during days 39–60. Shaded areas indicate 95% confidence intervals.

The GAMM analysis indicated that differences in feeding rates among breeds exhibit notable stage-dependent characteristics (Figure 4). Sex was initially included as a fixed effect in the GAMM but was not significant (*p* = 0.325) and did not improve model fit; therefore, it was excluded from the final model. Compared to the PS breed, DS, LT, and TY breeds showed significantly higher feeding rates during the early stage (approximately the first 20 days), but significantly lower rates during the mid-to-late stage (approximately days 30–60). Relative to the LT breed, the DS breed had a significantly lower feeding rate during the initial phase (approximately the first 10 days), but a significantly higher rate during the later growth period (approximately days 40–50). Compared to the TY breed, the DS breed exhibited a higher feeding rate during the first 10 days, but a significantly lower rate towards the end of the period (approximately days 50–60). Similar stage-dependent reversals were observed between LT and TY breeds: the LT breed had a higher feeding rate from days 0–10, but a significantly lower rate from days 39–60 compared to TY.

**Figure 5:**
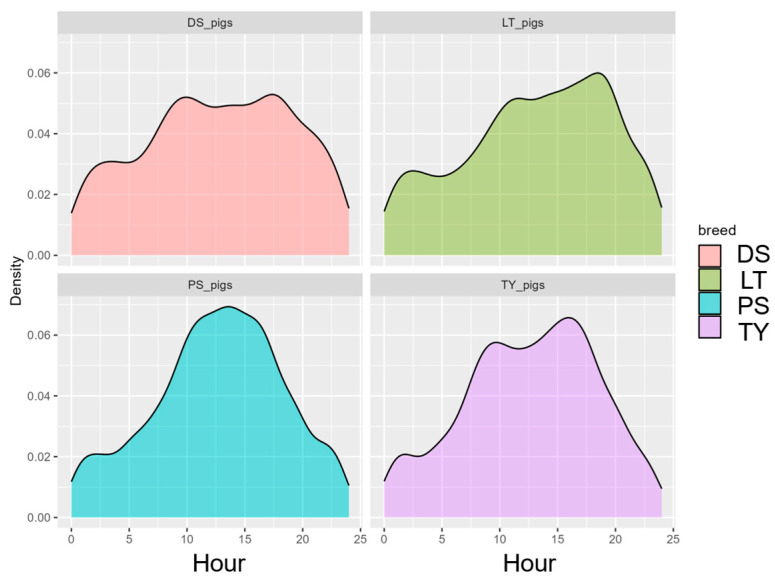
Diurnal distribution of feeding activity in DS, LT, PS, and TY pigs. Individuals from all four breeds exhibited a similar diurnal rhythm, with feeding activity peaking between 10:00 and 16:00. No significant differences in temporal distribution were observed among breeds (*p* > 0.05).

Furthermore, the study analyzed the temporal distribution of feeding activity across breeds (Figure 5). Individuals from all breeds displayed similar diurnal rhythms, with feeding activity peaking between 10:00 and 16:00, and no significant differences were observed among breeds (*p* > 0.05).

## 4. Discussion

This study systematically compared the growth performance, FCR variation patterns, and feeding behaviors of four indigenous pig breeds: DS, LT, PS, and TY breeds. Longitudinal body weight data were first used to establish growth models and compare growth rates across breeds. Subsequently, the stability of FCR estimation was evaluated across different time windows, upon which the effects of breed and growth stage on FCR were analyzed. Finally, GAMMs were employed to elucidate feeding rate and its temporal dynamics. The overall results indicate that significant differences exist among the four breeds in growth rate, feed efficiency, and feeding behavior dynamics, and that these differences are, to some extent, stage-dependent.

### 4.1. Breed as a Determinant of Growth Rate and Feed Efficiency

Under standardized basal diet conditions, inherent breed differences among the four Hunan indigenous pig breeds were the primary drivers of their divergent growth performance. The PS breed exhibited the greatest growth potential, with a daily gain (0.785 kg/day) significantly exceeding that of DS (0.485 kg/day), TY (0.450 kg/day), and LT (0.534 kg/day). Correspondingly, the FCR of the PS breed (3.98) was significantly lower than that of the other breeds, particularly DS (4.83) and TY (4.27), indicating a superior capacity for converting feed into body weight gain. This suggests that the physiological characteristics of the PS breed underlies its superior performance in both growth rate and feed efficiency. Interestingly, although daily gain in the TY breed did not differ significantly from that in DS and LT breeds, its FCR was notably superior to that of the DS breed. This implies that TY pigs may possess unique metabolic characteristics enabling them to achieve comparable growth levels with less feed consumption.

### 4.2. Dynamic Influence of Growth Stage on FCR and Rationale for Model Selection

FCR is a critical indicator for evaluating feed efficiency in pigs, but its calculation is susceptible to the length of the time window employed [39,40]. By comparing model performance across different time windows, this study found that model explanatory power increased as the time window extended from 7 to 21 days, suggesting that longer time windows mitigate the impact of short-term fluctuations on FCR estimation. This finding aligns with empirical observations in production settings, where short-term data are often influenced by daily feed intake fluctuations, environmental changes, and transient physiological states of individual animals [40,41,42].

When the time window was further extended to 30 days, model explanatory power declined instead. Analysis of the data structure indicates that this decline is primarily attributable to a reduction in the number of available observations and analyzable time points, rather than a biological change in FCR itself. This highlights the need to balance data stability and sample size when selecting an FCR calculation window in practical research. Ultimately, the 21-day window was chosen for analysis in this study, as it maintains high model explanatory power while ensuring adequate sample size for statistical analyses.

This study also revealed that FCR undergoes significant non-linear changes across growth stages. By comparing LM, LMM, and GAMM, we not only confirmed the main effect of breed but also uncovered the complexity of the time effect. Although both LM and LMM detected a linear increasing trend of FCR over time, the GAMM provided a more refined depiction of the dynamic fluctuations in FCR across growth stages for each breed. For instance, the FCR of the DS breed was significantly higher than that of the other breeds during the mid-growth phase, a pattern that deviates from the average trend revealed by linear models. Such non-linear trajectories indicate that a single average value across the growth period may obscure critical windows of FCR differences among breeds, underscoring the unique value of GAMM for precisely identifying these differences.

These findings carry important implications for both industry and research. From an industry perspective, the identification of stage-specific FCR patterns enables targeted nutritional interventions during critical windows, potentially reducing feed costs and improving profitability [25]. From a research standpoint, the results demonstrate that aggregate FCR values may mask breed-by-stage interactions, highlighting GAMM as a valuable analytical tool for future studies investigating the temporal dynamics of feed efficiency.

### 4.3. Breed-Specific Feeding Behavior Patterns and Growth Efficiency

Feeding behavior provides a direct indication of how pigs interact with feed resources and may contribute to variation in feed utilization efficiency among breeds [17,18]. In this study, breed differences were observed not only in average feeding rate but also in the temporal dynamics of feeding behavior.

The PS breed exhibited a higher baseline feeding rate than the DS breed, corresponding with its superior growth performance and the lowest FCR among the four breeds. These results suggest that differences in feeding behavior may contribute to breed-related variation in growth efficiency, although the underlying physiological and metabolic mechanisms require further investigation. The longitudinal analysis further demonstrated that breed differences in feeding rate were not constant throughout the experiment. During the early experimental phase, some breeds showed higher feeding rates, but were gradually overtaken by the PS breed in the mid-to-late period. This phenomenon may be related to environmental adaptation, social behaviors, and changing energy requirements across growth stages [43,44,45].

As the experiment progressed, pigs gradually acclimated to the rearing environment, potentially leading to adjustments in feeding behavior patterns and resulting in stage-dependent shifts in inter-breed differences. For example, DS pigs showed a higher feeding rate than LT pigs during the later growth period, but this increase in feeding rate was not associated with improved FCR. This indicates that feed intake behavior alone does not fully explain feed efficiency differences among breeds. The additional feed consumed by DS pigs may have contributed to increased maintenance requirements or other physiological processes rather than muscle deposition [46]. However, further studies integrating body composition and nutrient metabolism measurements are needed to clarify the underlying mechanisms.

Despite differences in feeding rate among breeds, all breeds exhibited similar diurnal feeding patterns, with feeding activity concentrated mainly between 10:00 and 16:00. This indicates that while breed differences influences feeding rate, diurnal rhythm may be more regulated by environmental factors such as circadian rhythms [47,48] and feeding management [49,50].

### 4.4. Practical Implications for Feeding and Management

The observed differences among indigenous pig breeds in growth performance, feed efficiency, and temporal patterns of feeding behavior provide potential insights for developing breed-specific feeding and management strategies. However, implications below should be interpreted cautiously, as the present study evaluated performance responses under a standardized feeding system and did not directly test breed-specific nutritional interventions. Further validation under different dietary and management conditions is required.

The PS breed exhibited relatively rapid growth, low FCR, and an increased feeding rate during the mid-to-late growth period. This period may represent an important stage for expressing its growth potential. Therefore, management strategies that ensure adequate nutrient supply and feeding access may be beneficial for supporting its production performance. Appropriate increases in dietary energy and protein levels, together with sufficient feeding space, may help fully leverage its growth potential and feed conversion efficiency [51,52]. Given the rapid feed intake observed in PS pigs, monitoring digestive health may also be important to minimize potential risks associated with high feeding rates.

The TY breed exhibited relatively lower daily gain but favorable feed efficiency, along with an adaptive period in feeding behavior. This pattern may indicate differences in behavioral adaptation or nutrient utilization strategies compared with the other breeds. Providing sufficient adaptation time during changes in environment or diet may therefore be beneficial. Management practices aim at improving early feed acceptance, such as maintaining stable feeding conditions and optimizing feed palatability, may help support feeding behavior establishment [52]; however, these approaches require further experimental evaluation.

The LT breed exhibits moderate daily gain and FCR, yet its feeding efficiency improves relatively slowly. Management strategies focusing on maintaining consistent feed intake and supporting nutrient utilization during later growth stages may potentially benefit production outcomes. Modifications in feed presentation, feeding frequency, or diet formulation could be explored as potential approaches [53,54], but their effects on LT pigs remain to be experimentally verified.

The DS breed exhibited relatively lower growth rate and feed efficiency, with higher FCR observed during the mid-growth period. These findings suggest that DS pigs may have different nutrient utilization characteristics compared with the other breeds. Therefore, optimization of dietary formulation, including nutrient balance and amino acid supply, may represent potential approaches for improving feed efficiency [55]. Nevertheless, breed-specific nutritional requirements should be determined through controlled feeding experiments before practical implementation.

### 4.5. Limitations and Future Directions

This study compared growth performance, feed conversion ratio (FCR), and feeding behavior among four indigenous pig breeds under standardized conditions. However, several limitations should be acknowledged.

The 60-day experimental period covered only part of the fattening stage and therefore did not capture complete growth trajectories or performance at later developmental stages. Longer-term studies are needed to evaluate breed differences throughout the entire growth period. In addition, the study included a limited number of animals from four indigenous breeds raised under a single management system. Consequently, the proposed 21-day observation window for FCR prediction was identified based on the current dataset and should be considered an empirically derived result. Its general applicability requires confirmation in larger populations and independent validation cohorts. Finally, this study focused on phenotypic differences and did not investigate the underlying biological mechanisms. Future studies integrating physiological and metabolic data may help explain the observed differences in growth performance and feed efficiency among indigenous pig breeds.

## 5. Conclusions

In summary, this study has established that the four Chinese indigenous pig breeds exhibit significant differences in growth performance, feed conversion efficiency, and feeding behavior during the fattening period. The PS breed exhibited superior growth performance and feed efficiency, which was associated with a higher baseline feeding rate compared with the other breeds. Both FCR and feeding rate undergo complex non-linear changes across growth stages in all breeds, with dynamic windows of inter-breed differences, highlighting the necessity of employing flexible models such as GAMM for analysis. These findings provide valuable insights into breed-specific characteristics and support the development of more targeted feeding and management strategies for indigenous pigs. Further studies integrating genetic, physiological, and metabolic analyses are needed to elucidate the mechanisms underlying breed differences in growth efficiency.

## Figures and Tables

**Table 1:** Composition and nutrient levels of the basal diet.

Ingredient	Content (%)	Calculated Nutrient Level	Content (%)
Corn	60.00	Digestible energy (DE, MJ/kg) ^b^	13.40
Soybean meal	19.00	Dry matter (DM, %) ^c^	95.95
Rice bran meal	12.00	Crude protein (CP, %) ^c^	19.37
Wheat flour	6.00	Ether extract (EE, %) ^c^	1.62
Dicalcium phosphate	0.40	Crude fiber (CF, %) ^c^	2.63
Limestone flour	0.30	Crude ash (CA, %) ^c^	8.08
Salt	0.30	Calcium (%)	0.26
2% Premix ^a^	2.00	Total phosphorus (TP, %) ^d^	0.44
Total	100.00	Available phosphorus (AP, %) ^d^	0.20

^a^ Purchased from Hunan Lifeng Biotechnology Co., Ltd. (Changsha, China). Provided per kilogram of diet: 19.8 mg CuSO_4_·5H_2_O; 0.20 mg KI; 400 mg FeSO_4_·7H_2_O; 0.56 mg NaSeO_3_; 359 mg ZnSO_4_·7H_2_O; 10.2 mg MnSO_4_·H_2_O; 5 mg vitamin K (menadione); 2 mg vitamin B_1_; 15 mg vitamin B_2_; 30 μg vitamin B_12_; 135 μg vitamin A; 2.75 μg vitamin D_3_; 0.45 μg vitamin E; 80 mg choline chloride. ^b^ DE of the experimental diet was estimated according to the prediction equation, based on the chemical composition (crude protein, ether extract, and fiber content) of the diet [27]. ^c^ Nutrient components were determined by chemical analysis in the laboratory. DM, C, EE, CF, and CA contents were measured according to AOAC proposed in 2005 methods: oven drying (DM, 930.15), Kjeldahl method (CP, 954.01), Soxhlet extraction (EE, 920.39), Weende fiber determination (CF, 978.10), and muffle furnace incineration (ash, 942.05). ^d^ Calcium content in each diet was calculated based on the inclusion rates of individual ingredients and their respective standard calcium values, sourced from NRC proposed in 2012. TP was obtained by weighted summation of phosphorus content from each ingredient; AP was estimated by multiplying TP by the phosphorus availability coefficient of each ingredient, with relevant parameters also based on NRC proposed in 2012 swine nutrient requirements.

**Table 2:** Observation summary of experimental animals.

Parameter	DS Pig	LT Pig	PS Pig	TY Pig
Number of pigs	12	12	12	12
Number of observations	731	708	729	702
Mean observation days per pig	60.9	59.0	60.8	58.5

**Table 3:** Growth models and estimated growth rates for different pig breeds.

Breed	Growth Model	Daily Gain (kg/day)	95% CI
DS pig	W = 56.46 + 0.485 d	0.485	0.409–0.561
LT pig	W = 59.00 + 0.534 d	0.534	0.458–0.610
PS pig	W = 61.10 + 0.785 d	0.785	0.709–0.861
TY pig	W = 56.30 + 0.450 d	0.450	0.374–0.526

CI: confidence interval, d: day, W: weight.

**Table 4:** Comparison of model performance across different FCR calculation time windows.

Time Window	Observations	Time Points	LM R^2^	LM Adjusted R^2^	LMM Marginal R^2^	LMM Conditional R^2^
7 days	296	9	0.161	0.129	0.152	0.329
10 days	219	6	0.308	0.281	0.295	0.361
14 days	189	5	0.26	0.231	0.253	0.259
17 days	149	4	0.393	0.368	0.384	0.384
21 days	129	3	0.478	0.457	0.467	0.506
30 days	87	2	0.406	0.377	0.395	0.395

**Table 5:** Comparison of FCR parameter estimates from three statistical models.

Parameter	LM	LMM	GAMM
Intercept (DS reference)	4.105 ***	4.101 ***	4.958 ***
LT vs. DS	−0.147 (*p* = 0.504)	−0.155 (*p* = 0.513)	−0.184 (*p* = 0.428)
PS vs. DS	−0.878 ***	−0.874 ***	−0.908 ***
TY vs. DS	−0.559 **	−0.556 *	−0.580 *
Time effect (21-day interval, linear trend)	0.848 ***	0.850 ***	-
sex	−0.194 (*p* = 0.199)	−0.196 (*p* = 0.234)	−0.206 (*p* = 0.195)

Note: * *p* < 0.05, ** *p* < 0.01, *** *p* < 0.001. In the GAMM, the time effect was modeled as a smooth function; therefore, linear trend estimates are not provided.

**Table 6:** Comparison of GAMM results.

Model	Parameters	AIC	BIC	Log Likelihood	χ^2^	df	*p*-Value
Null Model	6	−22,557	−22,521	11,284	-	-	-
Full Model	17	−22,628	−22,527	11,331	93.7	11	<0.001

**Table 7:** Parameter estimates for feeding rate by breed.

Parameter	Feeding Rate (kg/min)	Standard Error	t-Value	*p*-Value	Relative Change
Intercept (DS)	0.03037	0.00211	14.40	<0.001	Baseline
LT	0.03305	0.00267	1.01	0.314	+8.8%
PS	0.04267	0.00267	4.61	<0.001	+40.5%
TY	0.03275	0.00267	0.89	0.372	+7.8%
sex	−0.00186	0.00189	−0.98	0.325	−6.10%

Note: Due to equal sample sizes and balanced measurement structure across breeds, standard errors for breed dummy variables were consistent across models.

## Data Availability

The raw datasets are available from Zenodo: https://doi.org/10.5281/zenodo.20634963.

## Abbreviations

The following abbreviations are used in this manuscript:

AP	Available phosphorus
CA	Crude ash
AIC	Akaike information criterion
AR1	First-order autoregressive
ARMA	Autoregressive moving average
CF	Crude fiber
CP	Crude protein
CS	Compound symmetry
DE	Digestible energy
DM	Dry matter
DS	Dongshan
EE	Ether extract
FCR	Feed conversion ratio
GAMM	Generalized additive mixed model
LM	Linear model
LMM	Linear mixed model
LT	Longtan
PS	Pushi
RMSE	Root mean square error
SD	Standard deviation
TP	Total phosphorus
TY	Taoyuan
